# Advances in Biosensors, Chemosensors and Assays for the Determination of *Fusarium* Mycotoxins

**DOI:** 10.3390/toxins8060161

**Published:** 2016-05-24

**Authors:** Xialu Lin, Xiong Guo

**Affiliations:** Key Laboratory of Trace Elements and Endemic Diseases of National Health and Family Planning Commission, School of Public Health, Health Science Center, Xi’an Jiaotong University, No.76 Yan Ta West Road, Xi’an, Shanxi 710061, China; summer2047@yeah.net

**Keywords:** *Fusarium*, mycotoxins, biosensor, chemosensor, antibody, aptamer, molecularly imprinted polymer

## Abstract

The contaminations of *Fusarium* mycotoxins in grains and related products, and the exposure in human body are considerable concerns in food safety and human health worldwide. The common *Fusarium* mycotoxins include fumonisins, T-2 toxin, deoxynivalenol and zearalenone. For this reason, simple, fast and sensitive analytical techniques are particularly important for the screening and determination of *Fusarium* mycotoxins. In this review, we outlined the related advances in biosensors, chemosensors and assays based on the classical and novel recognition elements such as antibodies, aptamers and molecularly imprinted polymers. Application to food/feed commodities, limit and time of detection were also discussed.

## 1. Introduction

*Fusarium* mycotoxins are the general term of secondary metabolites produced by *Fusarium* species, the major families of which are fumonisins, trichothecenes, and zearalenone. Other emerging families of *Fusarium* mycotoxins include fusaproliferins, beauvercin, enniatins, butenolide, equisetin, moniliformin (MON) and fusarins [1]. They exist extensively in natural environment, especially in wheat, maize, rice, soybean and related byproducts. Fumonisins are mainly produced by *Fusarium (F.) verticillioides* and *F.* *proliferatum*. Approximately 15 different derivatives of fumonisins have been discovered, including fumonisin A1 (FA1), FA2, FB1, FB2, FB3, FB4, FC1, FC2, FC3, FC4 and FP1 [2]. The typical molecules of fumonisin compounds consist of a long hydroxylated hydrocarbon chain, with tricarballylic acid, methyl, and amino groups. Fumonisin B1 is the most toxic compound in this family, exhibiting hepato-, nephro-, immuno- and developmental toxicity in many animal species. It is also classified as Group 2B carcinogen (possibly carcinogenic to humans) by the International Agency for Research on Cancer [3]. Trichothecenes are mainly produced by *Fusarium*, *Myrothecium*, *Trichoderma*, *Trichothecium*, *Cephalosporium*, *Verticimonosporium*, and *Stachybotrys*. Over 200 mycotoxins are included in this family, all of which are sesquiterpene compounds [4]. According to the functional hydroxyl and acetoxy side groups’ variations, trichothecenes are divided into type A to type D. HT-2 toxin and T-2 toxin are the representatives in type A, nivalenol (NIV) and deoxynivalenol (DON) in type B. T-2 toxin can be toxic through skin intact, air exposure, and other exposure pathways. It mainly affected the highly proliferative cells, tissues and organs, such as thymus gland, lymphoid tissue, bone marrow, astrointestinal tract, and skin [5]. DON is the deoxygenated derivatives of NIV; it is also called a vomitoxin, highly cytotoxic, affecting intestinal, hematopoietic, immune, endocrine, and nervous systems. Zearalenone (ZEN) is produced by several *Fusarium* and *Gibberella* sepcies, such as *F. graminearum*, *F*. *culmorum*, *F*. *cerealis*, *F*. *equiseti*, and *F*. *verticillioides*. ZEN and its derivatives, such as α-zearalenol and β-zearalenol, are all potent estrogenic metabolites [6]. The reproductive system is the major toxicity target of this family toxin.

The contaminations of these *Fusarium* mycotoxins seriously influence the production of crops, the quality of agricultural products and animal feeds, and the safety of foods, and induce great economic losses and are great threats to human health. For this reason, the timely, rapid and accurate detection of the *Fusarium* mycotoxin contaminations in grain and its products, and the exposure level in human body are very important for risk monitoring and assessment. The classical analytical methods for *Fusarium* mycotoxins detections are the chromatographic techniques and chromatography-mass spectrometry linked techniques, which are based on the physical characteristics of toxins. These techniques need long and complicated sample pretreatment procedures, expensive instruments, skilled technicians and high determination cost, which are not suitable for the high-throughput detection of large samples. Based on the specific antigen–antibody reaction, traditional immunoassays, especially enzyme linked immuno-sorbent assay (ELISA) and lateral flow immunoassay (LFIA), are easy to perform and have been extensively used in the screening of *Fusarium* mycotoxins. However, there are some disadvantages, such as difficuly to automate the process, long testing time, or low sensitivity in different assays. There are some improvement, innovation and development on biorecognition assays. Meanwhile, novel developed optical, electrochemical, piezoelectric biosensors and chemosensors might be useful alternatives to solve these problems. In this review, we discussed these novel sensors and assays according to the recognition elements such as antibodies, aptamers and molecularly-imprinted polymers, and different detection signals.

## 2. Novel Biosensors and Assays Based on Antibodies

The antibody is the classical recognition element. Based on the specific immunological antibody–antigen reactions, many biosensors and assays have been developed, which are also called as immunosensors and immunoassays, respectively. Many immunosensors were developed from well-performed immunoassays. The transducer in immunosensors could directly or indirectly detect and measure the immunochemical reactions. According to the transducer types, immunosensors could be classified as optical, electrochemical, piezoelectric, and magnetic. Examples of the immunosensors and immunoassays for the detection of *Fusarium* mycotoxins are detailed in Table 1, Table 2, Table 3 and Table 4.

### 2.1. Optical Immunosensors and Immunoassays

Optical immunosensor and immunoassays are important kinds of immunosensors and immunoassays that are widely used for detection. The optical signals in the immunosensor and immunoassays system include light absorbance, light polarization and rotation, fluorescence, luminescence and phosphoresence. The fluorescence polarization immunoassay is a well-known rapid and sensitive detection assay. The main optical immunosensors included surface plasmon resonance immunosensor, fiber-optic immunosensor, and fluorescent array immunosensor. The advances of these immunoassays and immunosensors for the determination of *Fusarium* mycotoxins are discussed as follow.

Surface plasmon resonance (SPR) is a physical optics phenomenon at the interface between two different permittivity materials. The explanation and realization of SPR were extensively described by many reviews [92,93]. The SPR immunosensor was based on the detection of the mass concentration changes of analyte at the sensor surface. The first SPR immunosensor for FB1 detection was established by Mullett *et al.* in 1998 [8]. The specific antibodies were immobile on a gold film substrate and coupled to the glass slide. In the presence of different concentration FB1 in the sample cell, the resonance angle and reflected light intensity would be proportionally changed on the glass side and detected by the immunosensor [8]. Based on SPR, the rapid immunoassays for the DON [27,28,32,33], NIV [33] or T-2 toxin [47] detection were developed and improved subsequently, and applied in durum wheat, wheat products, maize-based baby foods, *etc.* SPR immunosensors for the simultaneous detection of two or more mycotoxins were also reported, such as “AFB1 (aflatoxin B1), ZEN, FB1 and DON” [79], “DON and ZEN” [84], and “DON, ZEN, T-2, OTA, FB1 and AFB1” [91] (see Table 4).

Fluorescence polarization immunoassay (FPIA) for *Fusarium* mycotoxins is based upon the change detection of fluorescence polarization signal before and after the competitive binding of fluorescently-labeled and unlabeled mycotoxin to the specific antibody. The fluorescently-labeled mycotoxin is called the FPIA tracer. It is in low molecular weight, and can rotate more rapidly, giving low fluorescence polarization signal. The signal is increased when the FPIA tracer binding to the antibody, which form a high molecular weight complex. After the extraction of samples, this assay is simple and easy to perform within a few minutes. These developed FPIAs were mostly applied to the detection in wheat or maize. The common fluoresceins and its derivatives for FPIA are fluorescein (FL), 4’-(aminomethyl) fluorescein (FL2), fluorescein isothiocyanate (FITC), 5- or 6- carboxy-fluorescein (CF), fluoresceinthiocarbamyl ethylenediame (EDF), 4’-(aminomethy) fluorescein hydrochloride (4’-AMF), fluoresceinthiocarbamyl hexamethylenediamine (HMDF) and [4,6-dichlorotriazine-2-yl]amino-fluorescein (DTAF). Maragos *et al.* reported the first application of FPIA in FB1 detection [10]. The FPIA tracer was labeled with 6-DTAF, and the assay got high cross-reactivity with FB2 (70%) and FB3 (77%) [10]. The FPIA with FB1-FITC and monoclonal antibody (mAb) 4B9 was found great cross-reactivity with FB2 (98.9%) and screened out for the simultaneous detection of FB1 and FB2 [16]. Rapid FPIAs for DON were also established using tracer, DON-FL [25,30,41] or DON-FL2 [26], for HT-2 and T-2 toxins using HT2-FL_1a_ [48], and for ZEN and its analogs using ZEN-FL [54,57], ZEN-HMDF [60], ZEN-4AMF [66], ZEN-EDF [68] or ZEN-AMF [68].

Besides FPIA, the fluoresceins were also applied to fluorescent biosensors. Carter *et al.* used the FITC labeled secondary antibody for ZEN detection [53]. Ngundi *et al.* labeled the anti-DON mAb with Cy5 bisfunctional dye for DON detection [29]. The fiber-optic immunosensor for FB1 measurement was developed and applied in maize samples [7,9,94]. In the study of Thompson *et al.*, the FB1 labeled with fluorescein, FB1-FITC, was firstly saturated with the FB1 mAbs bound to a core optical fiber [7]. In the presence of FB1, there was a competition of the mAb binding sites, resulting in a decrease of fluoresce signals [7]. Several fluorescent array biosensors were built for simultaneous detection of “FB and other toxins” [80], “OTA and DON” [81], or “OTA, DON, AFB1 and FB” [82]. In such array, the fluorescent labeled specific antibodies or different biotinylated mycotoxins were often immobilized on the waveguide; during the detection, the conjugated mycotoxins were completed with different concentration of free mycotoxins in the sample to bind to the antibodies [80].

Quantum dots (QDs) are small semiconductor nanoparticles with stable photoluminescence and great fluorescence quantum yields. In the study of Beloglazova *et al.*, the QD-loaded liposomes (phospholipids) were conjugate with ZEN as the fluorescent labels for the ZEN detection immunoassay [69]. Subsequently, the QDs were applied in the multiplex assay for simultaneous screening of DON, ZEN, AFB1, T-2 toxin and FB1 [86]. The sensitivity of the QD assay could be highly improved compared with the traditional fluorescent immunoassay or ELISA [69,86]. Quenchbody (Q-body) was a novel fluorescent technology. It contained a fluorophore in specific antibody domain, the fluorescence of which was quenched naturally. In sample analysis, the antigen was interacted with the Q-body and caused the fluorescence of Q-body to dose-dependently increase. Based on this, Yoshinari *et al.* developed an innovative immunosensor for DON determination using anti-DON Q-body [45].

There were other novel or modified optical immunosensors. Mirasoli *et al.* and Zangheri *et al.* applied the enzyme-catalyzed chemiluminescence (CL) in LFIAs for “FB1 + FB2” [12] or “AFB1 and FB1” [89] detection, the CL signals of which were measured by ultrasensitive cooled charge-coupled device sensor. Zhao *et al.* illustrated a novel chemiluminescent immunosensor for DON detection [40]. The DON antibodies were conjugated with the rotator ε-subunit of F_0_F_1_-ATPase. During the detection, the concentration of DON in samples was indirectly indicated by the ATP synthetic activity of F_0_F_1_-ATPase and measured by chemiluminesce through the luciferin-luciferase system [40]. Urraca *et al.* fabricated an automated flow-through fluorescent immunosensor for ZEN measurement, in which the ZEN in samples was competed with ZEN-HRP (horseradish-peroxidase) for the antibody binding site [56]. Nabok *et al.* combined the approaches of total internal reflection ellipsometry (TIRE) and immunoassay to develop the sensitive optical immunosensors for the detection of T-2 toxins [46,50] and ZEN [50]. Based on optical waveguide lightmode spectroscopy (OWLS) technique, Majer-Baranyi *et al.* established a direct and a competitive immunosensor for DON detection in spiked wheat samples [36]. Based on the biolayer interferometry (BLI) technology, Maragos *et al.* built an immunosensor for the DON detection in wheat flour [34]. In the presence of DON specific antibodies and the DON spike samples, there was a competition between the free and immobilized DON to bind to the antibodies [34]. When the materials bound to the tip of the fiber changed, the interference pattern of light reflected from the surface of this optical fiber was changed accordingly [34]. This BLI immunosensor was then modified by the amplification of the assay signal using the primary antibody labeled with colloidal gold [35]. Lv *et al.* fabricated an sensitive electrochemiluminescence (ECL) immunosensor with RuSi@Ru(bpy)_3_^2+^ for DON detection [43]. Nanoporous Co_3_O_4_ and Au were used to modify the electrode for electrode-driven luminescence process [43].

### 2.2. Electrochemical Immunosensors and Assays

The electrochemical immunosensor systems of mycotoxins were often composed of electrodes, binding layer with immobiling mycotoxins, primary antibody, secondary antibody labeled enzymes, reaction substrate and product, and transducer for measurements. The amperometric, potentiometric, conductimetric, voltammetric and impedimetric signals are often used in the electrochemical biosensors and assays to measure the mycotoxin affinity interactions to the analytical signal. Among them, amperometry was the most widely used one, and highly sensitive beyond the optical techniques.

Few electrochemical immunosensors for fumonisins detection were reported in the literatures. Kadir *et al.* developed the first electrochemical immunosensor for the detection of FB1 and FB2 in corn samples [11]. In this system, the ELISA for FBs was transferred to the gold screen-printed electrode surface, and the HRP enzyme label activity was detected by chronoamperometry using tetramethylbenzidine (TMB) and H_2_O_2_ substrate [11]. Jodra *et al.* explored a disposable electrochemical magnetoimmunosensor for FBs in the maize certified reference materials (CRMs) and beer samples [17]. In this sensor, the ELISA method of FBs were coupled with magnetic beads and transferred onto the surface of carbon screen-printed electrodes [17]. Masikini *et al.* illustrated an impedimetric fumonisin immunosensors based on the PdTe QDs-polymer-multi wall carbon nanotubes platform and applied it in the detection of corn CRMs [18]. In the FB1 electrochemical immunosensor of Yang *et al.*, the nanocomposite film of single-walled carbon nanotubes (SWNTs) and chitosan (CS) were used to modify the electrical conductivity on glass carbon electrode (GCE) [23]. The electrochemical signal was from the reaction of alkaline phosphatase in secondary antibody and the substrate α-naphthyl phosphate. Ezquerra *et al.* developed an eight-channel amperometric electrochemical array sensor for FB1 determination, and the antibodies were also fixed on the magnetic beads [21].

Several electrochemical immunosensors for DON detection were also reported. Romanazzo *et al.* developed an enzyme-linked-immunomagnetic-electrochemical assay for the detection of DON in wheat, breakfast cereal and baby food samples [31]. The immunomagnetic beads were coupled with eight magnetized screen-printed electrodes to form the electrochemical transducers. The recognition element of this assay, the Fab fragment against DON, showed high cross-reactivity with 3-Ac-DON [31]. In the electrochemical impedimetric immunosensor study of Wei *et al.*, the GCE used for DON analysis was modified with a composite made from fullerene (C_60_), ferrocene and the ionic liquid [37]. Kwon *et al.* fabricated the potentiometric immunosensor for DON analysis using the extended-gate metal oxide semiconductor field effect transistor [39]. Olcer *et al.* exhibited the detection of DON on a novel real-time amperometric electrochemical profiling platform with new electrode array, where Au quasi-reference electrode and shared reference/counter electrodes were comprised with the integrated microfluidics [42]. A label-free electrochemical impedimetric immunosensor for DON determination in wheat, roasted coffee and corm samples was fabricated by Sunday *et al.* using a gold nanoparticles-dotted 4-nitrophenylazo-functionalized graphene (AuNp/G/PhNO2) nanocatalyst [44].

Many studies have reported the electrochemical immunosensors for ZEN detection. Hervás *et al.* developed the ZEN electrochemical immunosensors using the antibody-coated magnetic beads for the detection of the maize CRMs and cereal-based baby food [61]. This immunosensor was modified using screen-printed electrodes [65]. The microfluidic chips [95] and electrokinetic magnetic beads [96] were also integrated into the electrochemical immunoassay for the ZEN determination to achieve *in situ* manipulation. Based on the GCE with multiwall carbon nanotubes, Panini *et al.* fabricated a ZEN immunosensor coupled with flow injection system for the detection in cereals [64]. In 2011 year study of Panini *et al.*, the microfluidic immunosensor of ZEN was coupled with the gold electrode and the antibodies were immobilized on the 3-aminopropyl-modified magnetic microspheres [67]. Feng *et al.* fabricated a non-enzymatic amperometric biosensor for ZEN analysis in pig feed using nitrogen-doped graphene sheets to amplify signal at the sensor platform [71]. Nanoporous PtCo alloy was used to label the secondary antibody and improve the electrocatalytic activity to H_2_O_2_ [71]. Liu *et al.* developed an ultrasensitive label-free amperometric immunosensor for the ZEN determination [72]. In this sensor, the Au@AgPt nanorattles with high electron transfer rate were used for the immobilization of antibodies, and the mesoporous carbon was used for the loading of the nanorattles with large specific surface area [72]. Regiart *et al.* developed a novel sensor for α-zearalanone (α-ZAL) determination by square-wave voltammertry on nanostructured functional platform [76]. The electrochemical sensors of FBs, trichothecenes and ZENs exhibited great sensitivity and simplicity, and should be encouraged to fabricate for simultaneous detection of mycotoxins.

### 2.3. Piezoelectric, and Other Immunosensors

The piezoelectric transducer is basically a mass balance, which could be used for the direct detection of the immunoreactions by mass alone, without any labels or secondary antibodies. The quartz crystal microbalance (QCM) was such an example, which consisted of a thin quartz disk with two gold electrodes. One of the electrodes was functionalized to sense the analyte. In the report of Spinella *et al.*, a QCM-based piezoelectric immunosensor for detection of AFB1, OTA and FB1 was tested, in which the antibodies were immobilized on the DSP-coated gold quartz crystals [87]. In a QCM impedance study of Nabok *et al.*, surprisingly large mass increase and film softening were measured as a result of specific binding between T-2 toxins and antibodies [46]. The suggested reason was the specific binding of large aggregates of hydrophobic molecules of T-2 toxins and the surrounding methanol solvent. However, Nabok *et al.* indicated that the biosensors based on the QCM for the quantification of T-2 toxins required further investigation [46]. Besides, there are a few other kinds of immunosensors. Mak *et al.* developed the magnetoresistive immunosensor for multiplex determination of AFB1, ZEN and HT-2 toxin [83]. The classic immunoassay was integrated into a magnetic nanotag detection platform [83]. Kong *et al.* developed a multi-immunochromatographic paper sensor for 20 types of mycotoxins detection, including ZENs, DONs, T-2 toxins, AFs, and FBs [90].

## 3. Biosensors, Chemosensors and Assays Based on Novel Recognition Elements

Based on the novel recognition elements, example of the biosensors and chemosensors for the detection of *Fusarium* mycotoxins are detailed in Table 1, Table 2, Table 3 and Table 4.

### 3.1. Aptamers Based Biosensors and Assays

Aptamers are artificial short single stranded oligonucleotides with 20–80 bases, either DNA or RNA, selected by a new combinatorial chemistry technology, the systematic evolution of ligands by exponential enrichment (SELEX). They can incorporate or integrate different targets, such as protein, enzyme, biotoxin, metallic ions, organic dyestuffs and pesticide, with high affinity and specificity through the spatial configuration complementary. The biosensor based on aptamer is also called as aptasensor.

Based on the FB1 aptamer screened by McKeague *et al.* [97], several recognition aptasensors and assays were developed. Wu *et al.* illustrated a novel fluorescence resonance energy transfer (FRET) system for FB1 analysis using quenchers, fluorophore and aptamers [13]. The sequences of molecular beacon (MB) was 5′-SH-(CH_2_)_6_-GCTCG CCAGCTTATTCAATT CGAGC-(CH_2_)_6_-H_2_N-3′, which is similar to part sequence of FB1 aptamers FB1 39. Complementary oligonucleotides to MB and FB1 aptamers was also synthesized, the sequence of which was 5′-AATTGAATAAGCTGG-3′. They attached the quenchers, gold nanoparticles (AuNPs) to the 5′ end of MB, and the fluorophore donors, NaYF4: Yb, Ho upconversion fluorescent nanoparticles (UCNPs) to the 3′ end of the MB. There is a hairpin-like stem-loop structure in the MB, where the fluorophore and quenchers were close, resulting in fluorescence quenching. In the first stage of analysis, the FB1 aptamers conjugated by the carboxylation-functionalized magnetic nanoparticles were hybridized with the complementary oligonucleotides. Then in presence of the samples with FB1, there were competitive bindings between FB1 and complementary oligonucleotides to aptamers. Due to the high affinity binding of FB1 and its aptamers, the complementary oligonucleotides were released, which could bound the loop of MB and form double stranded DNA, leading the fluorescence restoration. Finally, the concentration of FB1 was indirectly quantified by the fluorescence [13]. In sodium citrate buffer solution, the AuNPs were homogeneous and stable, showing red color. As the increase aggregation extent of AuNPs, red, purple, or blue color is exhibited in the solution. Wang *et al.* developed an aptasensor of FB1 with AuNPs [14]. One AuNPs solution was conjugated with a DNA1 sequence, 5′-SH-AATTGAATAAGCTGGTA-3′, which was complementary to part sequence of FB1 aptamers FB1 39. Another AuNPs solution was conjugated with DNA2 sequences, 5′-SH-TACCAGCTTATTCAATT-3′, which was complementary to DNA1. The DNA1-AuNPs solution was incubated with FB1 aptamers FB1 39 to make the sequence hybridization. In presence of FB1 solution, some FB1 aptamers were deviated from DNA1 sequence and bound to FB1 with high affinity. The liberative DNA1-AuNPs were then hybridized with DNA2-AuNPs, which made AuNPs close and changed the solution color. This assay indirectly detected the concentration of FB1 through its correlation with the color variation of AuNPs solution, the color of which could be quantified by the ultraviolet-visible spectrophotometry. Zhao *et al.* fabricated an ECL aptasensor for FB1 detection [15]. The nanoprobes of Au NPs and ionic iridium complex (novel ECL labels) were covalent with FB1 aptamers. The Au electrode was modified with DNA partial complementary (PC-DNA) to FB1 aptamer. With the concentration of FB1 increased in aptasensor, the ECL intensity would inverse proportionally decrease [15]. Chen *et al.* built a simple and sensitive FB1 aptasensor based on the microcantilever array sensors [19]. The reference microantilevers were only functionalized 6-mercapto-1-hexanol self-assembled monolayers, while the sensing microcantilevers were modified with SAMs of the FB1 aptamer FB1 39. In presence of FB1 sample solution, the sensing cantilevers could specifically combine the FB1 and lead to the deflection. The FB1 concentration could be indirectly quantified by this difference on the microcantilever biosensor. An impedimetric aptamer-based biosensor was developed to detect FB1 in maize samples [20]. The working electrode apF10/AuNPs/GCE was fabricated with GCE, modified by AuNPs on the surface, and conjugated with the FB1 aptamer F10. When the FB1 bound to the apF10/AuNPs/GCE electrode, there was higher inhibition of the electron transfer between the electrolyte buffer and this electrode, and larger resistance. The concentration of FB1 was indirectly related the change of electron transfer resistance (R_et_), and measured by the electrochemical impedance spectroscopy [20]. Shi *et al.* designed an electrochemical aptasensor for FB1 detection [22]. The GCE was modified by Au NPs, covalent with capture DNA and hybridized with FB1 aptamers. The graphene/thionine nanocomposites (GS-TH) were loaded to increase the electrochemical signal. In the presence of increasing FB1 concentration, the electrochemical signal would inversely decrease following the release of aptamers and GS-TH on GCE [22].

In 2013, Chen *et al.* isolated and identified a ZEN aptamer [70]. In this assay, the ZEN aptamer was labeled with biotin and coupled with streptavidin-coated magnetic beads. The ZEN in sample solutions was pre-concentrated by this ZEN aptamer, then separated and enriched by magnetic force and finally detected by fluorescence spectrophotometer [70]. This ZEN aptamer was supposed to be applied to biosensors. A high specificity and affinity aptamer of the monoclonal antibody against ZEN (mAb-ZEN) was identified by Wang *et al.* [75]. Moreover, an enzyme-linked oligonucleotide assay of ZEN was developed based on it. To detect the ZEN content, the mAb-ZEN was coated on microtiter plate. Then, ZEN solutions, the biotinylated mAb-ZEN aptamers, and HRP-conjugated streptavidin were successively incubated and washed. Finally, the TMB buffer was used for coloration, and the absorbance was measured by a microplate reader [75]. The LOD of DNA aptamer based sensors for FBs or ZENs determination could reach to “pg/mL” levels. However, there is a lack of the application of DNA aptamer on trichothecenes mycotoxin analysis.

Two simultaneous determination aptasensors of OTA and FB1 was developed. In the study of Wu *et al.* [85], two fluorophore donors, UCNPs of BaY_0.78_ F_5_:Yb_0.2_, Er_0.02_ and BaY_0.78_ F_5_:Yb_0.7_ were immobilized with OTA and FB1 aptamers, respectively [85]. Because of the strong π−π stacking effect, there was a spontaneous combination between the quenchers graphene oxide (GO) and the aptamers-UCNPs, resulting in the fluorescence quenching. When OTA and FB1 were involved, the nucleobases of aptamers were coupled with them instead of GO [85]. Its application on maize samples was conducted, and the measure results showed high correlation with the commercially available ELISA. In the study of Sun *et al.* [88], the surface of silica photonic crystal microphere was immobilized with the OTA or FB1 aptamers. Subsequently, the FITC labeled complementary DNA of related aptamers were used for hybridization. In the absence of OTA and FB1, the fluorescent intensities were high; in the presence of OTA and FB1, the related aptamers preferred to bind the target mycotoxins with high affinity and disassociated the complementary DNA, resulting in the decrease of fluorescence [88]. The measure results of its application on contaminated wheat, maize and rice samples were highly correlated with the classic ELISAs of OTA (*R*^2^ = 0.913) and FB1 (*R*^2^ = 0.993) [88].

### 3.2. Molecularly Imprinted Polymer Based Chemosensors

Molecularly imprinted polymers (MIPs) are artificial polymers with high affinity to specific molecules. Initially, the functional monomers were bound to the template molecules. Then, they were polymerized by crosslinkers. Finally, the template molecules were removed by physical or chemical methods, and left three-dimensional complementary cavities in the polymer matrix. Based on the technique of MIP and transducer, optical, electrical or quality chemosensor could be established for mycotoxin analysis.

Based on the MIP, Navarro-Villoslada *et al.* developed a chemosensor based on fluorescence displacement assay for ZEN analysis [58]. In the photo-polymerization of MIP, the cyclododecyl 2,4-dihydroxybenzoate (CDHB) was the synthetic mimics used as the templated molecule for ZEN; the 1-Allylpiperazine was the functional monomer. As the control, the non-imprint polymer was also synthesized without the template molecules. The fluorescent probe, 2,4- dihydroxybenzoic acid 2-[(pyrene-l-carbonyl)amino] ethyl ester (PARA) was tailor-made analogous to ZEN, and found high sensitivity to MIP and high sample throughput. This MIP/PARA-based fluorescence displacement sensor showed high sensitivity in ZEN solutions and high cross-reactivity with β-zearalenol [58]. In 2009 year, Choi *et al.* synthesized a molecularly imprinted polypyrrole (MIPPy) film on the Au SPR chip for ZEN detection [62]. Using a three-electrode electrochemical system, the functional monomer pyrroles were bound to template molecules ZEN, and electropolymerized on the Au SPR chip under the electrolytes of tetraethylammonium tetrafluoroborate. After the synthesis of this film, the ZEN and electrolytes in the polymeric matrix were removed by successive washing procedure in acetonitrile, methanol and chloroform. The films without the template molecules ZEN were also synthesized, called non-MIPPy. The SPR reflected intensities were measured on the MIPPy or non-MIPPy in the presence of ZEN. At the minimum SPR intensity, different concentration ZEN solutions were tested to determine the resonance angle shifts [62]. Using similar synthesis method, the MIPPy-SPR sensor for DON detection was also developed [38]. Gupta *et al.* developed a supersensitive chemical sensor for T-2 toxin analysis using MIP and SPR [49]. In the study of Gao *et al.*, the voltammetric electrochemical sensor for T-2 toxin determination was fabricated based on Fe^3+^-ion molecularly imprinted film [51]. This MIP sensor was successfully applied in cereals and human serum samples [51]. Based on the ionic liquid-stabilized CdSe/ZnS QDs, Fang *et al.* established a molecularly imprinted optosensing material (MIOM) for ZEN detection in the fluorescence sensors [73]. During the polymerization of MIOM, CDHB was used as the template molecules, and the modified CdSe/ZnS QDs were bound to the polymers as the fluorescent labels. The similar material without CDHB was also synthesized, called non-imprinted optosensing material. With the addition and binding of different concentration ZEN, the fluorescence intensity of MIOM would be accordingly quenched, and detected by spectrofluorometry. This MIOM of ZEN showed high recoveries for corn, rice and wheat flour samples [73].

### 3.3. Other Biosensors, Chemosensors and Assays

Beyond aptamers and molecularly imprinted polymers, there were a few other elements used for the recognition of *Fusarium* mycotoxins and applied in the sensors and assays, such as oxidation response on electrodes, β-cyclodextrin, yeast cells, and phages. Hsueh *et al.* illustrated an indirect electrochemical sensor for DON screening based on DON hydrolysis products in basic solutions, and employed it in rice samples [24]. Afzali *et al.* developed an electrochemical sensor for ZEN determination in beverage samples [74]. The oxidation response changes of ZEN were observed at multi-walled carbon nanotube modified carbon paste electrodes [74]. Toro *et al.* developed a novel electrochemical sensor for MON quantification in maize samples [52]. The electrochemical oxidation of MON was adsorbed at cysteamine self-assembled monolayers on gold electrodes and recorded by cyclic voltammograms [52]. Sadrabadi *et al.* designed a DNA based electrochemical biosensor for ZEN evaluation in wheat and milk samples [78]. The interaction between ZEN and double-stranded DNA was shown as the oxidation signal of adenine, and detected by differential pulse voltammetry at a pencil graphite electrode [78]. Dall’Asta *et al.* investigated the complexation mechanism between the ZEN and β-cyclodextrin, and reported the chemosensor for ZEN detection in maize samples [59]. Välimaa *et al.* developed a bioluminescent whole-cell biosensor for the detection of ZEN and its metabolites in milk products [63]. The modified firefly (*Photinus pyralis*) luciferase reporter gene (*luc*) was inserted into the engineered yeast cells under the control of a hormone-responsive element (HRE). The present estrogenic ligands in the cell were bound to the constitutively expressed hormone receptors and in turn, to the HRE, which could induce the *luc* gene expression. In the presence of d-luciferin substrate, different intensity luminescence was produced [63]. Andreu *et al.* reported a fluorometric–enzymatic assay for ZEN detection in corn samples [55]. The ZEN could react with β-NADH in the presence of the enzyme 3α-hydroxysteroid dehydrogenase, and the fluorescence intensity changes of β-NADH were measured [55]. The phage display mediated immunopolymerase chain reaction (PD-IPCR) is a novel and sensitive technology combined with immunoassay and PCR. A PD-IPCR for ZEN determination was developed and applied in cereals [77]. The variable domain of heavy-chain (VHH) anti-ZEN antibodies was used to produce anti-idiotypic VHH phages, which showed high affinity to anti-ZEN mAb. The phage particles of anti-idiotypic VHH phage clone Z1 was used to compete with the ZEN for antibody interaction and provided DNA templates for PCR. The fluorescence signals of PD-IPCR could sensitively reflect the concentration of ZEN [77].

## 4. Conclusions and Prospects

In the past two decades, there has been significant technological progress in optical, electrochemical, piezoelectric and other kinds of biosensors, chemosensors and assays for the determination of *Fusarium* mycotoxins, such as fumonisins, HT-2 toxin, T-2 toxin, nivalenol, deoxynivalenol and zearalenone. The sensitivity and efficiency were greatly improved by these novel sensors and assays. Besides classic antibodies, many novel recognition elements, such as aptamers and molecularly imprinted polymers, were usefully developed and applied in some mycotoxin detections. However, the novel exploitations to more mycotoxin families are still needed. The contamination level of mycotoxins in food and feed, and the exposure level in human body are both important issues for risk monitoring and assessment. More complex matrices, such as human plasma and urine, are needed to investigate. Meanwhile, the detection methods for multiple *Fusarium* mycotoxins are still very limited, and need more efforts to study.

## Figures and Tables

**Table 1:** Recent biosensors and assays for fumonisins determination.

Reference	Technique	Analyte	Element	Sample (Extraction)	LOD	Working Range	Detection Time
[7], 1996	Optical: fiber-optic	FB1	antibody	buffer and corn (80% methanol)	10 ng/mL	10–1000 ng/mL	NA
[8], 1998	Optical: SPR	FB1	antibody	NA	50 ng/mL	NA	<10 min
[9], 1999	Optical: fiber-optic	FB1	antibody	maize (75% methanol)	0.4–3.2 μg/g	NA	NA
[10], 2001	Optical: FPIA	FB1, FB2, FB3	antibody	maize (PBS)	0.5 μg/g	0.5–100 μg/g	<30 min
[11], 2010	EC: amperometric	FB1, FB2	antibody	corn (70% methanol)	5 ng/mL	1–1000 ng/mL	NA
[12], 2012	Optical: CL	FB1, FB2	antibody	maize flour (PBS)	2.5 ng/mL	2.5–500 ng/mL	25 min
[13], 2013	Optical: FRET	FB1	aptamer	maize (70% methanol)	0.01 ng/mL	0.01–100 ng/mL	NA
[14], 2013	Optical	FB1	aptamer	beer	125 pg/mL	125–1500 pg/mL	NA
[15], 2014	Optical: ECL	FB1	aptamer	NA	0.29 ng/mL	NA	NA
[16], 2015	Optical: FPIA	FB1, FB2	antibody	maize (40% methanol)	53.6–290.6 ng/g	108.0–13166 ng/g	30 min
[17], 2015	EC: amperometric	FB1, FB2, FB3	antibody	maize-based foodstuffs (acetonitrile:PBS (50:50)), beer	0.33 ng/mL	0–1000 ng/mL	NA
[18], 2015	EC: impedimetric	FB1, FB2, FB3	antibody	corn (70% methanol)	0.46 pg/L	7–49 pg/mL	NA
[19], 2015	microcantilever array	FB1	aptamer	NA	33 ng/mL	0.1–40 μg/mL	NA
[20], 2015	EC: impedimetric	FB1	aptamer	maize (20% methanol)	2 pM	0.1 nM–100 μM	40 min
[21], 2015	EC: amperometric	FB1	antibody	cereal samples (70% methanol)	0.58 ng/mL	0.6–54 ng/mL	NA
[22], 2015	EC: amperometric	FB1	aptamer	wheat	1 pg/mL	1–106 pg/mL	NA
[23], 2015	EC: amperometric	FB1	antibody	corn (50% acetonitrile)	2 pg/mL	0.01–1000 ng/mL	NA

Note: FB: Fumonisin B; LOD: limit of detection; NA: not available; SPR: Surface plasmon resonance; FPIA: Fluorescence polarization immunoassay; EC: electrochemical; CL: chemiluminescence; FRET: fluorescence resonance energy transfer; ECL: electrochemiluminescence.

**Table 2:** Recent biosensors, chemosensors and assays for the determination of trichothecenes and other mycotoxins.

Reference	Technique	Analyte	Recognition Element	Sample (Extraction)	LOD	Working Range	Detection Time
Deoxynivalenol (DON) and Nivalenol (NIV)							
[24], 1999	EC: amperometric	DON	redox reactions	rice samples (85% acetonitrile)	9.1 μM/0.24 ppm	0.32–32 ppm	NA
[25], 2002	Optical: FPIA	DON, 15-Ac-DON	antibody	wheat	NA	NA	NA
[26], 2002	Optical: FPIA	DON, 3-Ac-DON	antibody	wheat, maize (PBS)	0.1 ng/g	NA	5 min
[27], 2002	Optical: SPR	DON	antibody	wheat (10% methanol, 6% polyvinylpyrrolidone)	2.5 ng/mL	0.13–10.0 μg/mL	15 min
[28], 2003	Optical: SPR	DON	antibody	wheat (80% acetonitrile)	NA	2.5–30 ng/mL	NA
[29], 2006	Optical: fluorescent, array	DON	antibody	cornmeal, cornflakes, wheat, barley, oats and indoor air (75% methanol)	0.2 ng/mL in buffer, 50 ng/g in oats, 4 ng/L in air	NA	NA
[30], 2006	Optical: FPIA	DON	antibody	durum wheat kernels, semolina, and pasta	NA		
[31], 2010	EC: amperometric	DON, 3-Ac-DON	Fab fragment	wheat, breakfast cereal and baby-food (84% acetonitril)	0.063 ng/mL	100–4500 ng/mL	NA
[32], 2010	Optical: SPR	DON, 3-AcDON	antibody	durum wheat, wheat products, and maize-based baby foods (40% methanol)	6–57 ng/g	250–2000 ng/g	6.5 h/20 samples
[33], 2010	Optical: SPR	NIV, DON	antibody	wheat (water)	NIV:0.1 μg/g; DON: 0.05 μg/g	NA	NA
[34,35], 2011, 2012	Optical: BLI	DON	antibody	wheat flour (0.02 M phosphoric acid)	0.10, 0.09 μg/g	NA	NA
[36], 2011	Optical: OWLS	DON	antibody	wheat (60% acetonitrile)	NA	0.01–50 ng/mL	NA
[37], 2011	EC: impedimetric	DON	antibody	food samples (water)	0.3 pg/mL	0.001–0.3 ng/mL	NA
[38], 2011	Optical: SPR	DON, 3-ADON, 15-ADON	MIP	standard solution	>1 ng/mL	0.1–100 ng/mL	NA
[39], 2011	EC: potentiometric	DON	antibody	PBS	0.1 ppm	NA	NA
[40], 2012	Optical: CL	DON	antibody	NA	0.1 ng/mL	0.1–10^5^ ng/mL	20 min
[41], 2014	Optical: FPIA	DON	antibody	wheat bran and whole-wheat flour (PBS)	120 ng/g	NA	10–15 min
[42], 2014	EC: amperometric	DON	antibody	wheat (water)	6.25 ng/mL	6.25–250 ng/mL	NA
[43], 2015	Optical: ECL	DON	antibody	wheat flour	1 pg/mL	0.005–100 ng/mL	NA
[44], 2015	EC: impedimetric	DON	antibody	wheat, roasted coffee and corn (water)	0.3 ng/mL	6–30 ng/mL	NA
[45], 2015	Optical: Q-body	DON	antibody	wheat (distilled water)	6 ng/mL in wheat	0.3–3000 ng/mL	NA
**T-2 toxin and moniliformin (MON)**							
[46], 2007	Optical: TIRE	T-2 toxin	antibody	NA	NA	0.15 ng/mL–100 μg/mL	NA
[47], 2010	Optical: SPR	T-2 and HT-2 toxins	antibody	breakfast cereal, wheat and baby food (40% methanol)	6–57 ng/g	250–2000 ng/g	9 min
[48], 2011	Optical: FPIA	HT-2 and T-2 toxins	antibody	wheat (90% methanol)	8 ng/g	NA	10 min
[49], 2011	Optical: SPR	T-2 toxin	MIP	NA	0.1 fM (0.05 pg/mL)	NA	NA
[50], 2011	Optical: TIRE	T-2 toxin	antibody	grain-food samples (acetonitrile)	<0.1 ng/mL	NA	NA
[51], 2014	EC: voltammetric	T-2 toxin	MIP	cereals and human serum (water and methanol, or chloroform)	0.15 μg/g	1.1 nM–2.1 μM	>25 min
[52], 2016	EC: voltammetric	MON	oxidation	maize (84% acetonitrile)	0.83 nM	1 nM–100 nM	NA

Note: LOD: limit of detection; NA: not available; EC: electrochemical; FPIA: fluorescence polarization immunoassay; SPR: surface plasmon resonance; BLI: biolayer interferometry; OWLS: optical waveguide lightmode spectroscopy; MIP: Molecularly imprinted polymer; CL: chemiluminescence; ECL: electrochemiluminescence; Q-body: Quenchbody; TIRE: total internal reflection ellipsometry.

**Table 3:** Recent biosensors, chemosensors and assays for zearalenone determination.

Reference	Technique	Analyte	Recognition Element	Sample (Extraction)	LOD	Working Range	Detection Time
[53], 2000	Optical: fluorescent	ZEN	antibody	NA	5 ng/mL	NA	60 min
[54], 2004	Optical: FPIA	ZEN and its metabolites	antibody	maize (84% acetonitrile)	110 ng/g	NA	10 min
[55], 2004	Optical: fluorescent	ZEN	enzymes	corn (a mixture of methanol or acetonitrile and water and NaCl)	NA	1–10 μg/mL	NA
[56], 2005	Optical: HRP, Flow-though	ZEN	antibody	corn, wheat, and swine feed samples	0.007 ng/mL	0.019–0.422 ng/mL	NA
[57], 2006	Optical: FPIA	ZEN	antibody	maize	0.04 g/mL	0.01 to 1 g/mL	NA
[58], 2007	Optical: fluorescent	ZEN	MIP	NA	25 μM	NA	NA
[59], 2008	Optical: fluorescent	ZEN	β-cyclodextrin	maize (H_2_O-CH_3_CN mixture (20:80, *v/v*))	50 ng/g	NA	NA
[60], 2009	Optical: FPIA	ZEN	antibody	cereal products (70% methanol and 4% NaCl)	137 ng/g	150–1000 ng/g	<2 min
[61], 2009	EC: amperometric	ZEN	antibody	maize, baby food, cereal (acetonitril:methanol (50:50) or 75% acetonitrile)	0.011ng/mL	NA	NA
[62], 2009	Optical: SPR	ZEN	MIP	corn (70% methanol)	0.3 ng/g	0.3-3000 ng/mL	NA
[63], 2010	Optical: bioluminescent	ZEN and its metabolites	yeast cells	milk (90% milk and 10% ethanol)	2 nM for ZEN	NA	<3 h
[64], 2010	EC: amperometric	ZEN	antibody	corn silage (70% methanol)	0.77 ppb	0–500 ppb	NA
[65], 2010	EC: potentiometric	ZEN	antibody	baby food (75% acetonitrile)	7 pg/mL	NA	NA
[66], 2011	Optical: FPIA	ZEN and its metabolites	antibody	corn (60%–75% methanol)	77 ng/g	100–5000 ng/g	3 min
[50], 2011	Optical: TIRE	ZEN	antibody	aqueous solutions	0.1 ng/mL	NA	NA
[67], 2011	EC: amperometric	ZEN	antibody	feedstuffs (70% methanol)	0.41 ng/g	NA	30 min
[68], 2012	Optical: FPIA	ZEN	antibody	ground grain (60% methanol)	3 ng/mL	NA	NA
[69], 2013	Optical: QD	ZEN	antibody	NA	0.02–0.6 ng/g	NA	NA
[70], 2013	Optical	ZEN	aptamer	beer	0.785 nM	3.14 nM–31.4 μM	NA
[71], 2013	EC: amperometric	ZEN	antibody	pig feed (70% methanol)	2.1 pg/mL	0.005–25 ng/mL	NA
[72], 2014	EC: amperometric	ZEN	antibody	NA	1.7 pg/mL	0.005–15 ng/mL	NA
[73], 2014	Optical: fluorescent	ZEN	MIP	cereal crops (acetonitrile)	0.002 μM	0.003–3.12 μM	NA
[74], 2015	EC: amperometric	ZEN	oxidation	malt beverage samples	0.58 ng/mL	2.0–50 ng/mL	NA
[75], 2015	ELONA	ZEN	aptamer	corn (70% methanol)	0.01 ng/mL	0.03–2.5 ng/mL	NA
[76], 2015	EC: voltammetric	α-ZAL	antibody	bovine serum	16 pg/mL	0.05–50 ng/mL	12 min
[77], 2016	PD-IPCR	ZEN	phage particles	corn, wheat and rice (60% methanol)	6.5 pg/mL	0.01–100 ng/mL	NA
[78], 2016	EC: voltammetric	ZEN	dsDNA	milk and wheat (85%acetonitrile for wheat)	5 pg/mL	0.008–20 ng/mL	NA

Note: ZEN: zearalenone; α-ZAL: α-zearalanone; LOD: limit of detection; NA: not available; FPIA: fluorescence polarization immunoassay; HRP: horseradish-peroxidase; MIP: Molecularly imprinted polymer; EC: electrochemical; SPR: surface plasmon resonance; TIRE: total internal reflection ellipsometry; QD: Quantum dot; ELONA: enzyme-linked oligonucleotide assay; PD-IPCR: phage display mediated immuno-PCR.

**Table 4:** Recent biosensors, chemosensors and assays for the simultaneous determination of *Fusarium* and other mycotoxins.

Reference	Technique	Analyte	Element	Sample (Extraction)	LOD (Working Range)	DT
[79], 2003	Optical: SPR	AFB1, ZEN, FB1, DON	antibody	NA (90% acetonitrile)	LOD: 0.01–50 ng/g	25 min
[80], 2003	Optical: fluorescent, array	FB1, ricin, cholera toxin, *etc.*	antibody	NA	FB: 250ng/mL	NA
[81], 2006	Optical: fluorescent, array	OTA, DON	antibody	barley, cornmeal, wheat and maize (75% methanol)	LOD: (ng/g) DON: 1–180; OTA: 1–85.	NA
[82], 2006	Optical: fluorescent, array	OTA, DON, AFB1 and FB	antibody	NA	LOD: AFB1: 0.3 ng/mL	15 min
[83], 2010	Magnetoresistive	AFB1, ZEN, HT-2	antibody	NA	LOD: 50 pg/mL	
[84], 2011	Optical: SPR	DON, ZEN	antibody	maize and wheat (acetonitrile–water–formic acid (84:16:1))	LOD: (ng/g) DON: 68–84; ZEN: 40–64	14 min
[85], 2012	FRET	OTA, FB1	aptamer	maize	(ng/mL) OTA: 0.02 (0.05–100); FB1: 0.1 (0.1–500)	NA
[86], 2014	Optical: QD	DON, ZEN, AFB1, T-2, FB1	antibody	wheat and maize samples (80% methanol)	LOD: (ng/g) SAM FISA: DON: 3.2, ZEN: 0.6, AFB1: 0.2, T-2: 10, FB1: 0.4; DAM FISA: ZEN: 1.8, AFB1: 1.	NA
[87], 2014	Piezoelectric: QCM	AFB 1, OTA, FB1	antibody	standard solution	Range: 0.5–10 ppb	NA
[88], 2014	Optical:	OTA, FB1	aptamer	rice, corn, and wheat (60% methanol)	(pg/mL) OTA: 0.25 (10–1000); FB1: 0.16 (1–1000)	NA
[89], 2015	Optical: CL	FBs, AFB1	antibody	maize flour (PBS)	(ng/mL) FB1:0.6 (0.6–1500); AFB1: 0.15 (0.15–50)	30 min
[90], 2016	Optical	ZENs, DONs, T-2 toxins, AFs, FBs, *etc.*	antibody	cereal food samples	(ng/g) ZENs: 0.04–0.17, DONs: 0.06–49, T-2 toxins: 0.15–0.22, AFs: 0.056–0.49, FBs: 0.53–1.05	20 min
[91], 2016	Optical: SPR	DON, ZEN, T-2, OTA, FB1, AFB1	antibody	barley (80% methanol)	(ng/g) DON: 26, ZEN: 6, T-2: 0.6, OTA: 3, FB1: 2, AFB1: 0.6	NA

Note: AF: aflatoxin; AFB1: aflatoxin B1; ZEN: zearalenone; FB: fumonisin B; DON: deoxynivalenol; OTA: ochratoxin A; HT-2: HT-2 toxin; T-2: T-2 toxin; LOD: limit of detection; NA: not available; SPR: surface plasmon resonance; FRET: fluorescence resonance energy transfer; QD: Quantum dot; FLISA: fluorescent immunosorbent assay; SAM: single-analyte multiplex; DAM: double-analyte multiplex; QCM: quartz crystal microbalance; CL: chemiluminescence.

## Abbreviations

The following abbreviations are used in this manuscript:

AFB	aflatoxin B
AuNP	gold nanoparticle
4’-AMF	4’-(aminomethy) fluorescein hydrochloride
BLI	biolayer interferometry
CF	5- or 6- carboxy-fluorescein
CL	chemiluminescence
CRM	certified reference material
CS	chitosan
CDHB	cyclododecyl 2,4-dihydroxybenzoate
DAM	double-analyte multiplex
DON	deoxynivalenol
DTAF	[4,6-dichlorotriazine-2-yl]amino-fluorescein
EC	electrochemical
ECL	electrochemiluminescence
EDF	fluoresceinthiocarbamyl ethylenediame
ELISA	enzyme linked immuno-sorbent assay
ELONA	enzyme-linked oligonucleotide assay
*F.*	*Fusarium*
FA	fumonisin A
FB	fumonisin B
FC	fumonisin C
FITC	fluorescein Isothiocyanate
FL	fluorescein
FL2	4’-(aminomethyl) fluorescein
FLISA	fluorescent immunosorbent assay
FRET	fluorescence resonance energy transfer
FP	fumonisin P
FPIA	fluorescence polarization immunoassay
GCE	glass carbon electrode
GO	graphene oxide
HMDF	fluoresceinthiocarbamyl hexamethylenediamine
HRE	hormone-responsive element
HRP	horseradish-peroxidase
LFIA	lateral flow immunoassay
LOD	limit of detection
mAb	monoclonal antibody
MB	molecular beacon
MIP	Molecularly imprinted polymer
MIOM	molecularly imprinted optosensing material
MIPPy	molecularly imprinted polypyrrole
MON	moniliformin
NA	not available
NIV	nivalenol
OTA	ochratoxin A
OWLS	optical waveguide lightmode spectroscopy
PARA	2,4- dihydroxybenzoic acid 2-[(pyrene-l-carbonyl)amino] ethyl ester
PC-DNA	DNA partial complementary
PD-IPCR	phage display mediated immuno-PCR
QCM	quartz crystal microbalance
QD	quantum dot
Q-body	quenchbody
SAM	single-analyte multiplex
SELEX	systematic evolution of ligands by exponential enrichment
SPR	surface plasmon resonance
SWNTs	single-walled carbon nanotubes
TIRE	total internal reflection ellipsometry
T-2	T-2 toxin
TMB	tetramethylbenzidine
UCNP	upconversion fluorescent nanoparticles
VHH	variable domain of heavy-chain
α-ZAL	α-zearalanone
ZEN	zearalenone
